# Humidity resilient ionogels for joint pressure monitoring

**DOI:** 10.1093/nsr/nwae412

**Published:** 2024-11-15

**Authors:** Yadong Xu, Wei Gao

**Affiliations:** Andrew and Peggy Cherng Department of Medical Engineering, California Institute of Technology, USA; Andrew and Peggy Cherng Department of Medical Engineering, California Institute of Technology, USA

Hydrogels have garnered substantial attention in the field of soft electronics due to their flexibility and tissue-like elasticity, making them ideal for interfacing with biological tissues and organs [1]. However, they often suffer from low electrical conductivity and are prone to drying out over time due to water evaporation [2,3], limiting their long-term functionality. Ionogels, which address these limitations, have emerged as a promising alternative. These materials incorporate polymer networks swollen in ionic liquids or are formed by polymerizing monomers within ionic liquids [4]. Ionic liquids endow ionogels with unique properties—such as ionic conductivity, non-volatility, and high thermal and electrochemical stability—making them suitable for a broad spectrum of applications, including wearable electronics, energy storage, and sensing technologies [5,6]. Nonetheless, a persistent challenge with ionogels has been their hygroscopic nature, especially in humid or wet conditions, as in implantable biomedical devices [7]. In such conditions, ionogels can absorb water, which disrupts their ionic pathways and can compromise their structural integrity—posing a critical concern for implantable applications where stable performance is required in the high-humidity and dynamic environment of the human body.

**Figure 1.**
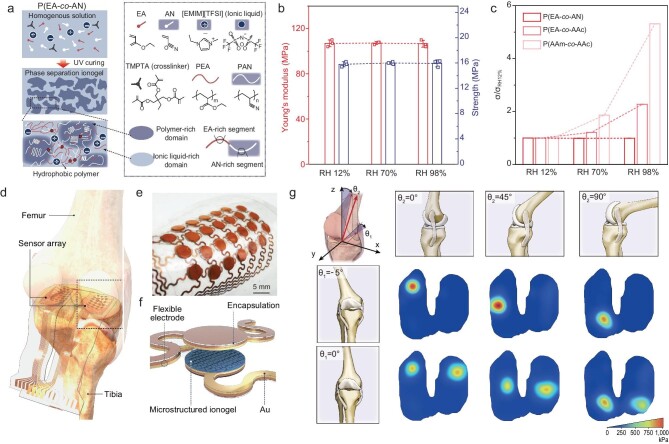
(a) A schematic of the fabrication process of humidity-resilient P(EA-*co*-AN) ionogel from *in situ* phase separation. (b and c) Mechanical (b) and electrical (c) resilience of the ionogel to various relative humidity levels of up to RH 98%. P(EA-*co*-AAc) and P(AAm-*co*-AAc) are also compared in (c) as hydrophilic counterparts. (d) A schematic of intra-articular pressure monitoring. (e) A photograph of the flexible iontronic pressure-sensing array. (f) An exploded illustration of the sensor array. (g) Real-time monitoring of intra-articular pressure with a live sheep model. Adapted with permission from Ref. [10].

Humidity-resilient ionogels have the potential for expanding the scope of wearable and implantable bioelectronics [8,9], notably enhancing the durability, longevity, performance, and functionality of iontronic sensors. Shi *et al.* introduce a non-hygroscopic, humidity-insensitive ionogel-based iontronic sensor array specifically engineered for robust intra-articular pressure sensing [10]. The strategy utilizes acrylonitrile (AN) and ethyl acrylate (EA) as monomers and 1-ethyl-3-methylimidazolium bis(trifluoromethylsulfonyl)imide ([EMIM][TFSI]) as the ionic liquid (Fig. 1a). Initially, the monomers are fully miscible with the ionic liquid, forming a clear solution; however, upon polymerization, phase compatibility differences lead to an *in-situ* phase separation, resulting in two hydrophobic phases: a liquid-rich polyethyl acrylate (PEA) phase that enhances ionic conductivity and stretchability, and a liquid-poor polyacrylonitrile (PAN) phase that provides substantial toughness. The resulting P(EA-*co*-AN) ionogel, composed entirely of hydrophobic phases, exhibits high water-contact angles (99° on flat surface and 120° on microstructured surface), enabling excellent resistance to humidity. Quantitative tests further highlight its humidity-insensitive performance, with minimal variations in Young's modulus, tensile strength, and electrical conductivity even under 98% relative humidity for 30 minutes—substantially outperforming hydrophilic ionogels (Fig. 1b and c).

Leveraging humidity-resistant ionogels as active layers, the researchers developed a pressure sensor array with humidity-insensitive performance, achieving a sensitivity of ∼2.46 kPa⁻¹ under 98% humidity across a wide pressure range (0–2 MPa). This stable and reliable sensing system is well suited for applications involving exposure to bodily fluids. Moreover, its thin, soft, and stretchable form factor enables a close fit with the joint, enabling strain-insensitive sensing under various deformations (Fig. 1d**–**f). In the study, the authors demonstrated the sensor array's applicability in a live sheep model for real-time monitoring of intra-articular pressure without the need for trial molds typically required during traditional intra-articular pressure detection during total knee replacement surgeries. This system enables real-time mapping of joint surface pressure and holds potential for extension to other articular joints (Fig. 1g).

Shi and colleagues present a breakthrough in developing a hydrophobic, humidity-resilient ionogel with broad potential applications in biomedical implants, skin-interfaced bioelectronics, soft robotics, and environmental sensors. Its durability and flexibility make it especially suitable for intra-articular sensors and prosthetics, where stable performance in moist environments is critical. Nonetheless, the demands of long-term, continuous biosignal monitoring highlight the need for further research into the ionogel's stability and biocompatibility. Achieving scalable, cost-effective manufacturing will also be essential for clinical deployment. While the ionogel demonstrates humidity stability, it may face challenges in extreme high-temperature conditions; future research could focus on enhancing its thermal resilience. Addressing these challenges could expand the ionogel's practical applications, establishing it as a versatile foundation for advanced sensing technologies.
